# Lipoprotein Lipase as a Candidate Target for Cancer Prevention/Therapy

**DOI:** 10.1155/2012/398697

**Published:** 2011-10-19

**Authors:** Shinji Takasu, Michihiro Mutoh, Mami Takahashi, Hitoshi Nakagama

**Affiliations:** Division of Cancer Development System, National Cancer Center Research Institute, 5-1-1 Tsukiji, Chuo-ku, Tokyo 104-0045, Japan

## Abstract

Epidemiological studies have shown that serum triglyceride (TG) levels are linked with risk of development of cancer, including colorectal and pancreatic cancers, and their precancerous lesions. Thus, it is assumed that serum TG plays an important role in carcinogenesis, and the key enzyme lipoprotein lipase (LPL), which catalyzes the hydrolysis of plasma TG, may therefore be involved. Dysregulation of LPL has been reported to contribute to many human diseases, such as atherosclerosis, chylomicronaemia, obesity, and type 2 diabetes. Recently, it has been reported that *LPL* gene deficiency, such as due to chromosome 8p22 loss, *LPL* gene polymorphism, and epigenetic changes in its promoter region gene, increases cancer risk, especially in the prostate. In animal experiments, high serum TG levels seem to promote sporadic/carcinogen-induced genesis of colorectal and pancreatic cancers. Interestingly, tumor suppressive effects of LPL inducers, such as PPAR ligands, NO-1886, and indomethacin, have been demonstrated in animal models. Moreover, recent evidence that LPL plays important roles in inflammation and obesity implies that it is an appropriate general target for chemopreventive and chemotherapeutic agents.

## 1. Introduction

A high-calorie diet and low physical activity, part of the so-called “Westernization” of lifestyle, are associated with elevated incidences of the breast, colon, liver, pancreas, and prostate cancers. Moreover, they are also linked with the risk of obesity, type 2 diabetes, and dyslipidemia. The World Cancer Research Fund and American Institute for Cancer Research have evaluated causal relationships between body fat and cancer and provided strong evidence for roles in such as colorectum and pancreas cancers [1]. In Japan, overweight and obesity (body mass index ≥25) are reported to be associated with cancers of specific organs, such as the colorectum (male), postmenopausal breast (female), and the liver in individuals positive for hepatitis C virus infection [2–4]. 

Greater body fatness is a major risk factor for the metabolic syndrome, which presents as a combination of symptoms, such as dyslipidemia (elevated triglyceride (TG) levels or low high-density lipoprotein (HDL) cholesterol), elevated blood pressure, and elevated fasting glucose levels. Hypertriglyceridemia is associated with the risk of colon cancer in Japanese men (HR = 1.71) and being overweight with the risk of breast cancer (HR = 1.75) [5]. In addition, most epidemiological studies, including our own, have consistently showed that serum TG levels are associated with the risk of colorectal adenoma, a precursor lesion of colorectal cancer [6–11]. Thus, it is assumed that serum TG could play an important role in carcinogenesis and that the key enzyme lipoprotein lipase (LPL), which catalyzes the hydrolysis of plasma TG, may also be involved. In this paper, we focus on the roles of LPL in cancer development and further discussed possible approaches to cancer prevention/therapy.

## 2. Function, Structure, and Gene Regulation of LPL

### 2.1. Functions and Structure of LPL

LPL plays an important role in lipid metabolism as an enzyme responsible for hydrolysis of the TG component in circulating chylomicrons and very-low-density lipoprotein (VLDL) via binding with apolipoprotein C2 [12, 13]. Thus, lowering or deficiency of LPL expression is associated with hyperlipidemia [14, 15]. The LPL enzyme itself is composed of two structurally distinct regions. The amino-terminal domain is responsible for catalysis with a catalytic center formed by three amino acids (Ser^132^, Asp^156^, and His^241^). The carboxy-terminal domain of LPL is required for its binding to the lipoprotein substrate [3, 16–18]. 

### 2.2. LPL Gene Expression and Its Regulation

The human *LPL *gene is located on chromosome 8p22 and composed of 10 exons [19]. LPL is ubiquitously expressed in the whole body, but especially in the adipose tissue and the skeletal muscle [20, 21] and is regulated by hormonal and inflammatory stimuli, such as insulin [22, 23], glucocorticoid [24, 25], adrenaline [26], tumor necrosis factor (TNF)-*α* [27, 28], transforming growth factor (TGF)-*β* [29], and interleukin (IL)-1*β* [27].

The expression of LPL is controlled transcriptionally and posttranscriptionally. Basal promoter activity has been shown to be regulated by Oct-1 and the NF-Y binding motifs [30, 31], and the 5′-CCTCCCCC-3′ motif, which interacts with Sp1 and Sp3 [32]. Induction of *LPL *gene transcription is mediated by the peroxisome proliferator response element (PPRE) and the responsible element which binds to sterol regulatory element-binding protein (SREBP) [33, 34]. The effect of insulin on *LPL* expression is an example of posttranscriptional control, the hormone being suggested to increase *LPL *mRNA levels via mRNA stabilization [23, 35].

## 3. Relationship between LPL and Cancer: Human Studies

### 3.1. Loss of LPL and Resultant Common Disease

LPL has been reported to play key roles in many human diseases, such as atherosclerosis, obesity, type 2 diabetes, chylomicronaemia, Alzheimer's disease, and cachexia [15]. Especially, *LPL* gene deficiency is the cause of type I hyperlipoproteinemia (familial hyperchylomicronemia) [36]. Homozygous deficiency of *LPL *in humans is rare, but heterozygous deficiency is observed in around 3% of people with various ethnic backgrounds [37, 38]. Although these individuals have elevated serum levels of TG and decreased HDL cholesterol [39], it is not clear whether they are at increased risk of atherosclerosis, ischemic heart disease, type 2 diabetes, and cancer. There is a report that the *LPL *S447X mutation is associated with a higher risk of pancreatic calcification and steatorrhea in hyperlipidemic pancreatitis [40]. Since LPL provides fatty acids to the tissues and fatty acids evoke insulin resistance, *LPL* gene deficiency could affect glucose metabolism. However, whether heterozygous *LPL* deficiency reduces plasma glucose levels or not is still controversial. One paper described reduction of plasma glucose levels, but two others observed no effects as compared with LPL intact humans [41–43]. On the other hand, it has been reported that patients with poorly controlled diabetes frequently have dyslipidemia due to defects in LPL enzyme activity [44].

### 3.2. Effects of Chromosome 8p22 Loss and LPL Gene Polymorphisms on Cancer Risk

Alteration in genomic DNA, such as point mutations and deletions/amplifications or epigenetic changes such as CpG island hypermethylation and histone modification, can induce abnormal gene expression, which in the case of tumor suppressor genes or oncogenes could eventually lead to carcinogenesis. The human *LPL* gene has been mapped to chromosome 8p22 and previous studies on loss of heterozygosity (LOH) in colorectal tumors suggested that a putative tumor suppressor gene may lie within the short arm of chromosome 8, that is, 8p22-p21.3. Loss of 8p23.1-22 is also reported to be an important stage in initiation or promotion of hepatocellular carcinoma development and may also be the most frequent chromosomal alteration in prostate cancer [45]. It has been found that deletion of *LPL* is observed in 68% (52/76) of localized prostate cancers by FISH analysis [46]. It has further been reported that chromosomal region 8p23.1-8p21.1 may harbor one or more important prostate-cancer-susceptible loci based on linkage analyses in 159 hereditary prostate cancer families [47, 48]. To date, several new candidate cancer-susceptible genes have been cloned to 8p22, such as *deleted in breast cancer 2* (*DBC2*), *leucine zipper tumor suppressor 1* (*LZTS1*), *deleted in liver cancer 1* (*DLC1*), and *mitochondrial tumor suppressor 1* (*MTUS1*) [49–52]. Thus, cancer-susceptible genes mapped close to the *LPL* gene could be affected by *LPL* gene deletion, and exert combined effects in promoting carcinogenesis. 

Moreover, an *LPL *Ser447stop polymorphism has been shown to be associated with prostate cancer risk [53] and the *LPL* gene is commonly methylated in prostate tumors [54]. *LPL* promoter CpG island methylation has been revealed in 45% of LPL-deleted tumors and in 22% of LPL-retaining tumors [54]. Biallelic inactivation of *LPL* by chromosomal deletion and promoter methylation may thus contribute to prostate tumorigenesis, but information is lacking regarding pancreatic cancer.

## 4. Relationship between LPL and Cancer: Animal Studies

### 4.1. Dyslipidemia Observed in Cancer-High-Susceptibility Animal Models

Elevated serum TG has been shown to promote carcinogen-induced colon carcinogenesis, and rats with hypertriglyceridemia such as the Zucker obese and Nagase analbuminemic strains and F344 rats fed a high-fat diet are all known to be more sensitive to carcinogen treatments than rats with normal serum lipid levels [55–57]. 

In the case of mice, the *Apc* ^1309^ (C57BL/6J^*Apc*/*Apc*Δ1309^) [58] and Min (C57BL/6-*Apc* ^*Min*⁡/+^) animal models of human familial adenomatous polyposis (FAP) feature development of large numbers of intestinal polyps and hypertriglyceridemia [59, 60]. Although no significant differences between *Apc* ^1309^ mice and wild-type mice were observed at 6 weeks of age, the average serum TG value in the former at 12 weeks was obviously increased almost 10-fold (~600 mg/dL) over that at 6 weeks. Similar increase of TG levels (~400 mg/dL) was observed in Min mice at 15 weeks compared to 8 weeks of age (Table 1). Along with TG elevation, mRNA levels of *LPL* in the liver and small intestine of *Apc* ^1309^ and Min mice were suppressed. Of note, other lipogenic genes, such as *FAS *and *stearoyl-CoA desaturase-1*, *β*-oxidation genes like *acyl-CoA oxidase* and *carnitine palmitoyl transferase 1*, and gluconeogenesis genes, exemplified by *phosphoenolpyruvate carboxykinase*, demonstrated no variation from wild-type mouse expression. 

Obese KK-*A^y^* mice were found to be highly susceptible to azoxymethane- (AOM-) induced colorectal aberrant crypt foci (ACF) and colorectal carcinoma development compared to lean C57BL/6J mice [61]. Surprisingly, colorectal carcinomas developed within a very short-term period, 19 weeks, after AOM injection. The number of total ACF in KK-*A^y^* mice was around 70/mouse and almost 8 times higher than that in lean C57BL/6J mice. The incidences of adenomas and adenocarcinoma were 84% and 88%, respectively, in KK-*A^y^* mice, far higher than the 8% and 4% in C57BL/6J values. KK-*A^y^* mice exhibit abdominal obesity, hypertriglyceridemia, and hyperinsulinemia at the time of ACF and tumor development. At 13 weeks of age, the average serum levels of TG, total cholesterol, and free fatty acids of KK-*A^y^* mice undergoing AOM treatment were 484.1 mg/dL, 101.6 mg/dL, and 1,796 mEq/L, respectively (Table 1). It is interesting that hepatic *LPL *mRNA levels were also suppressed in KK-*A^y^* mice compared with C57BL/6J mice. Moreover, serum proinflammatory adipocytokines, such as IL-6, leptin, and plasminogen activator inhibitor-1 (Pai-1), were elevated. Importantly, expression of pro-inflammatory adipocytokine mRNAs such as for IL-6, leptin, monocyte chemotactic protein (MCP)-1, Pai-1 and TNF-*α* was significantly increased in the visceral fat tissue; in contrast, that for adiponectin was decreased.

Tanaka et al. have developed a novel colitis-related colorectal carcinogenesis model, using AOM plus dextran sodium sulfate (DSS), a colitis-inducing agent [64]. In this model (AOM + 2% DSS in ICR mice), numerous colorectal adenocarcinomas occur within a short-term period and the serum TG levels demonstrate increase to about 134, 175 and 159 mg/dL at 5, 10, and 20 weeks, respectively [62] (Table 1). 

Injection of *N*-nitrosobis(2-oxopropyl)amine (BOP) into Syrian golden hamsters is known to induce pancreatic ductal adenocarcinomas, with a histology very similar to typical human pancreatic ductal adenocarcinomas. Moreover, associated genetic mutations, that is, K-*ras* point mutations and *p16* aberrant methylation/homozygous deletions, are found in common in both hamster and human lesions. Interestingly, Syrian golden hamsters exhibit a hypertriglyceridemic state, almost 300 mg/dL at 6 weeks of age, even when not fed a high-fat diet [63] (Table 1). Also, in the case of this animal model, a low activity of LPL could be one of the causes of hypertriglyceridemia, activity of this enzyme in the liver being only 20% and 30%, respectively, of the values in C57BL mice and F344 rats.

## 5. Tumor Suppressive Effects of LPL Inducers 

**Figure 1:**
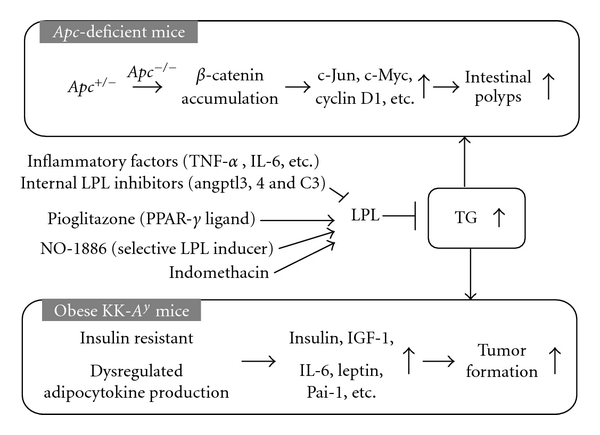
Involvement of triglycerides in animal intestinal carcinogenesis models. Angptl-3,4: angiopoietin-like protein-3,4; IGF-1: insulin like growth factor-1; IL-6: interleukine-6; LPL: lipoprotein lipase; Pai-1: plasminogen activator inhibitor-1; PPAR: peroxisome proliferator-activated receptor; TG: triglyceride; TNF-*α* **: **tumor necrosis factor-*α.*

Pioglitazone, {(±)-5-[4-[2-(5-ethyl-2-pyridyl)ethoxy]benzyl]thiazolidine-2,4-dione monohydrochloride}, is a potent peroxisome proliferator-activated receptor (PPAR)*γ* ligand with a weak binding affinity for PPAR*α*. In the promoter region of the* LPL *gene, there exists a PPRE, and pioglitazone treatment successfully induced LPL expression in the liver and intestinal epithelial cells in *Apc*-deficient mice. The total numbers of polyps in the groups treated with 100 and 200 ppm pioglitazone in the *Apc* ^1309^ were reduced to 67% of the value in the untreated control group [59] (Table 2). With another *Apc*-deficient model, Min mice given 100–1600 ppm pioglitazone for 14 weeks showed decrease of intestinal polyps to 63–9% of the control number [60] (Table 2 and Figure 1). 

Pioglitazone possesses other functions rather than just simply inducing LPL, such as causing cell growth arrest and apoptosis. Thus, data regarding LPL selective inducers are necessary for determining the relationship between hypertriglyceridemia and intestinal carcinogenesis. NO-1886, 4-[(4-bromo-2-cyanophenyl)carbamoyl] benzylphosphonate, chemically synthesized at Otsuka Pharmaceutical Factory [67] is one useful tool for clarifying this issue. Using a reporter gene assay, NO-1886 demonstrated no PPAR agonistic activity, unlike bezafibrate and pioglitazone [68]. 

Administration of 400 and 800 ppm NO-1886 also significantly decreased the total number of intestinal polyps to 48% and 42% of the untreated control value, respectively, in Min mice, along with causing marked increase in *LPL *mRNA levels in the liver and the small intestine. Moreover, treatment with NO-1886 also significantly decreased the numbers of colon polyps [65] (Table 2, Figure 1). 

In the case of BOP-treated hamsters, pioglitazone has been demonstrated to improve hyperlipidemia and suppress ductal adenocarcinoma development. The incidences of ductal adenocarcinoma in the BOP plus 800 ppm pioglitazone and BOP alone groups were 38% and 80%, and the multiplicities were 0.55 and 1.37, respectively [63] (Table 2). Expression levels of hepatic LPL mRNA were elevated by treatment with 800 ppm pioglitazone. Moreover, quantitative real-time RT-PCR assays demonstrated almost 1.7-fold higher mRNA levels of LPL than that of pioglitazone-nontreated hamsters.

Indomethacin is a conventional nonsteroidal anti-inflammatory drug which has long been clinically employed to improve inflammation. It has demonstrated potent chemopreventive activity against intestinal tumor development in animal models, and a clinical trial in FAP patients also showed reduction in intestinal polyp development [69, 70]. We earlier reported that indomethacin suppresses intestinal polyp formation in Min mice together with ameliorating the hyperlipidemic state by regulating LPL, other lipid metabolic factors and inflammatory pathways [66]. Reduction of serum TG levels was 90% in Min mice with 10 ppm indomethacin treatment and higher than that with 400 ppm pioglitazone (83%) observed in our other previous study [59, 60]. The PPAR*γ* agonistic activity of indomethacin is reported to be 50 times weaker than that of troglitazone, a well-established PPAR*γ* agonist [71]. These results indicate that functions other than agonistic activity of indomethacin are responsible for its strong lipid-lowering effects (Figure 1).

## 6. Involvement of LPL in Inflammation, Obesity, and Others

### 6.1. LPL and Inflammation and Apoptosis

In addition to the lipid modifying function of LPL, two different mechanisms might be involved in *LPL* influence on carcinogenesis. The first involves anti-inflammatory action of LPL. It has been reported that LPL suppresses TNF-*α*- and interferon (IFN)-*γ*-evoked inflammation-related gene expression in endothelial cells through inactivation of transcription factor nuclear factor kappa B (NF-*κ*B) [72]. Conversely, TNF-*α*, IFN-*γ*, IL-1*β*, IL-6, and leukemia inhibitory factor (LIF) decrease LPL activity.

It is well known that cyclooxygenase-2 (COX-2) is markedly elevated in human colon cancers, in AOM-treated rats, and in intestinal polyps of *Apc*-deficient mice. COX-2 is in fact thought to play important roles in both cancer cell proliferation and angiogenesis. Experiments conducted to clarify the mechanisms of NO-1886 effects on colon carcinogenesis revealed that the expression levels of mRNA for COX-2, in DLD-1 human colon cancer cells, were reduced under conditions of TGF*α* stimulation. On the other hand, there was no obvious change in the mRNA levels for COX-1 and inducible nitric oxide synthase (iNOS). The results obtained by RT-PCR analysis were also confirmed by *β*-gal reporter gene assay in DLD-1 cells [65]. Consistent with the *in vitro* data, administration of 400 and 800 ppm NO-1886 reduced COX-2 mRNA levels in normal parts of small intestine of Min mice at 20 weeks of age [65]. In addition, NO-1886 ameliorates and induces regression of experimental steatohepatitis through increasing LPL activation and suppression of proinflammatory agents, such as TNF-*α*, IL-6, and COX-2 [73]. Recently, mice lacking *angiopoietin-like protein family 4* (*Angptl4*), which is the inhibitor of LPL, showed a severe and lethal phenotype characterized by fibrinopurulent peritonitis, ascites, intestinal fibrosis, and cachexia in response to a saturated fat diet [74].

The second mechanism is modification of the apoptosis pathway by LPL activation. Phosphatase type 2C*β* activation by unsaturated fatty acids has been demonstrated to induce apoptosis [75]. Unlike ester bodies of fatty acids, free fatty acids have cytotoxic effects *in vitro* and the products produced by hydrolysis of plasma TG may be implicated in such an apoptotic effect.

### 6.2. LPL and Obesity

Given the importance of LPL for lipid metabolism, its activity would be expected to be intimately involved in obesity effects and development of the metabolic syndrome. A large number of studies in rodents and humans have revealed that obesity results in increased LPL activity in adipose tissue [15, 35, 76–78]. Interestingly, LPL is regulated in opposite directions in adipose tissue and muscle. Feeding increases adipose LPL activity with a corresponding decrease in muscle LPL activity [35, 79]. Exercise stimulates LPL activity in the muscle and leads to increase fatty acid oxidation [80]. In an animal study, NO-1886 suppressed high-fat diet-induced fat accumulation in rats due to the increase of muscle LPL activity [81].

## 7. Conclusion

Targeting LPL activity or expression levels for development of reagents against cancer seems particularly challenging, because LPL is expressed ubiquitously and plays essential roles in maintaining homeostasis in the body. Data from LPL homozygous knockout mice, which die within one day of birth, underline its importance. However, appropriate suppression of serum TG levels could be achieved by using drugs, even if the number of selective inducers of LPL is limited. Thus, it might be important to develop selective LPL inducers or search for agents focusing on the aspect of “drug repositioning” to obtain the tools for investigating correlation between LPL and cancer. It should be borne in mind that LPL is inhibited by intrinsic factors, such as angptl3, angptl4, and C3 (Figure 1). These could clearly be candidate target molecules for development of LPL inducers. Considering that LPL activity has impact on obesity and metabolic syndrome, its targeting may also affect the regulation of adipocytokines, which may also be involved in carcinogenesis. Further investigations are warranted to clarify the importance of LPL and to accumulate evidence as to the worthiness as a target for cancer chemopreventive and chemotherapeutic agents.

## Figures and Tables

**Table 1:** Summary of animal models with dyslipidemia and cancer high susceptibility.

Animal	Strain	Age (week-old)	Serum TG (mg/dL)	Treatment	Tumor	Reference
Mouse	*Ap* *c* ^1309^ (C57BL/6J^*Apc*/*Apc*Δ1309^)	12	~600	—	Intestinal adenoma	[59]
	Min (C57BL/6-*Apc* ^*Min*⁡/+^)	15	~400	—	Intestinal adenoma	[59, 60]
	KK-*A^y^*	19	481	AOM	Colon cancer	[61]
	ICR	20	159	AOM + DSS	Colon cancer	[62]
Syrian golden hamster	—	6	300	BOP	Pancreatic cancer	[63]

**Table 2:** Summary of tumor suppressive effects of LPL inducers in animal models.

Agent	Dose	Animal model	Value to the untreated control group	Reference
Pioglitazone	200 ppm	*Ap* *c* ^1309^	67%	[59]
	1600 ppm	Min	9%	[60]
	800 ppm	BOP-treated hamster	40%	[63]
NO-1886	800 ppm	Min	42%	[65]
Indomethacin	10 ppm	Min	25%	[66]

## References

[1] World Cancer Research Fund/American Institute for Cancer Research (2007). *Food, Nutrition, Physical Activity, and the Prevention of Cancer: A Global Perspective*.

[2] Otani T, Iwasaki M, Inoue M (2005). Body mass index, body height, and subsequent risk of colorectal cancer in middle-aged and elderly Japanese men and women: Japan public health center-based prospective study. *Cancer Causes and Control*.

[3] Iwasaki M, Otani T, Inoue M, Sasazuki S, Tsugane S (2007). Body size and risk for breast cancer in relation to estrogen and progesterone receptor status in Japan. *Annals of Epidemiology*.

[4] Inoue M, Kurahashi N, Iwasaki M (2009). Metabolic factors and subsequent risk of hepatocellular carcinoma by hepatitis virus infection status: a large-scale population-based cohort study of Japanese men and women (JPHC Study Cohort II). *Cancer Causes and Control*.

[5] Inoue M, Noda M, Kurahashi N (2009). Impact of metabolic factors on subsequent cancer risk: results from a large-scale population-based cohort study in Japan. *European Journal of Cancer Prevention*.

[6] Kono T, Ikeda N, Yanai F, Yamamoto M, Shigematsu T (1990). Serum lipids and colorectal adenoma among male self-defence officials in Northern Kyushu, Japan. *International Journal of Epidemiology*.

[7] Bird CL, Ingles SA, Frankl HD, Lee ER, Longnecker MP, Haile RW (1996). Serum lipids and adenomas of the left colon and rectum. *Cancer Epidemiology Biomarkers and Prevention*.

[8] Manus B, Adang RP, Ambergen AW, Brägelmann R, Armbrecht U, Stockbrügger RW (1997). The risk factor profile of recto-sigmoid adenomas: a prospective screening study of 665 patients in a clinical rehabilitation centre. *European Journal of Cancer Prevention*.

[9] Park SK, Joo JS, Kim DH, Kim YE, Kang D, Yoo KY (2000). Association of serum lipids and glucose with the risk of colorectal adenomatous polyp in men: a case-control study in Korea. *Journal of Korean Medical Science*.

[10] Shinomiya S, Sasaki J, Kiyohara C (2001). Apolipoprotein E genotype, serum lipids, and colorectal adenomas in Japanese men. *Cancer Letters*.

[11] Otani T, Iwasaki M, Ikeda S (2006). Serum triglycerides and colorectal adenoma in a case-control study among cancer screening examinees (Japan). *Cancer Causes and Control*.

[12] Miller AL, Smith LC (1973). Activation of lipoprotein lipase by apolipoprotein glutamic acid. Formation of a stable surface film. *Journal of Biological Chemistry*.

[13] Eisenberg S, Rachmilewitz D (1975). Interaction of rat plasma very low density lipoprotein with lipoprotein lipase rich (postheparin) plasma. *Journal of Lipid Research*.

[14] Gehrisch S (1999). Common mutations of the lipoprotein lipase gene and their clinical significance. *Current Atherosclerosis Reports*.

[15] Mead JR, Irvine SA, Ramji DP (2002). Lipoprotein lipase: structure, function, regulation, and role in disease. *Journal of Molecular Medicine*.

[16] Winkler FK, D’Arcy A, Hunziker W (1990). Structure of human pancreatic lipase. *Nature*.

[17] Van Tilbeurgh H, Roussel A, Lalouel JM, Cambillau C (1994). Lipoprotein lipase. Molecular model based on the pancreatic lipase X-ray structure: consequences for heparin binding and catalysis. *Journal of Biological Chemistry*.

[18] Bengtsson-Olivecrona G, Olivecrona T, Jornvall H (1986). Lipoprotein lipases from cow, guinea-pig and man. Structural characterization and identification of protease-sensitive internal regions. *European Journal of Biochemistry*.

[19] Wong H, Schotz MC (2002). The lipase gene family. *Journal of Lipid Research*.

[20] Semenkovich CF, Chen SH, Wims M, Luo CC, Li WH, Chan L (1989). Lipoprotein lipase and hepatic lipase mRNA tissue specific expression, developmental regulation, and evolution. *Journal of Lipid Research*.

[21] Goldberg IJ (1996). Lipoprotein lipase and lipolysis: central roles in lipoprotein metabolism and atherogenesis. *Journal of Lipid Research*.

[22] Ong JM, Kirchgessner TG, Schotz MC, Kern PA (1988). Insulin increases the synthetic rate and messenger RNA level of lipoprotein lipase in isolated rat adipocytes. *Journal of Biological Chemistry*.

[23] Semenkovich CF, Wims M, Noe L, Etienne J, Chan L (1989). Insulin regulation of lipoprotein lipase activity in 3T3-L1 adipocytes is mediated at posttranscriptional and posttranslational levels. *Journal of Biological Chemistry*.

[24] Ong JM, Simsolo RB, Saffari B, Kern PA (1992). The regulation of lipoprotein lipase gene expression by dexamethasone in isolated rat adipocytes. *Endocrinology*.

[25] Fried SK, Russell CD, Grauso NL, Brolin RE (1993). Lipoprotein lipase regulation by insulin and glucocorticoid in subcutaneous and omental adipose tissues of obese women and men. *Journal of Clinical Investigation*.

[26] Carneheim C, Nedergaard J, Cannon B (1988). Cold-induced beta-adrenergic recruitment of lipoprotein lipase in brown fat is due to increased transcription. *The American Journal of Physiology*.

[27] Doerrler W, Feingold KR, Grunfeld C (1994). Cytokines induce catabolic effects in cultured adipocytes by multiple mechanisms. *Cytokine*.

[28] Mackay AG, Oliver JD, Rogers MP (1990). Regulation of lipoprotein lipase activity and mRNA content in rat epididymal adipose tissue in vitro by recombinant tumour necrosis factor. *Biochemical Journal*.

[29] Friedman G, Ben-Yehuda A, Ben-Naim M, Matsa D, Stein O, Stein Y (1995). Effect of transforming growth factor-*β* on lipoprotein lipase in rat mesenchymal heart cell cultures. *Biochimica et Biophysica Acta*.

[30] Previato L, Parrott CL, Santamarina-Fojo S, Brewer HB (1991). Transcriptional regulation of the human lipoprotein lipase gene in 3T3-L1 adipocytes. *Journal of Biological Chemistry*.

[31] Morin CL, Schlaepfer IR, Eckel RH (1995). Tumor necrosis factor-*α* eliminates binding of NF-Y and an octamer- binding protein to the lipoprotein lipase promoter in 3T3-L1 adipocytes. *Journal of Clinical Investigation*.

[32] Yang WS, Deeb SS (1998). Sp1 and Sp3 transactivate the human lipoprotein lipase gene promoter through binding to a CT element: synergy with the sterol regulatory element binding protein and reduced transactivation of a naturally occurring promoter variant. *Journal of Lipid Research*.

[33] Schoonjans K, Gelman L, Haby C, Briggs M, Auwerx J (2000). Induction of LPL gene expression by sterols is mediated by a sterol regulatory element and is independent of the presence of multiple E boxes. *Journal of Molecular Biology*.

[34] Schoonjans K, Peinado-Onsurbe J, Lefebvre AM (1996). PPAR*α* and PPAR*γ* activators direct a distinct tissue-specific transcriptional response via a PPRE in the lipoprotein lipase gene. *EMBO Journal*.

[35] Braun JEA, Severson DL (1992). Regulation of the synthesis, processing and translocation of lipoprotein lipase. *Biochemical Journal*.

[36] Havel RJ, Gordon RS (1960). Idiopathic hyperlipemia: metabolic studies in an affected family. *The Journal of Clinical Investigation*.

[37] Benlian P

[38] Fisher RM, Mailly F, Peacock RE (1995). Interaction of the lipoprotein lipase asparagine 291 → serine mutation with body mass index determines elevated plasma triacylglycerol concentrations: a study in hyperlipidemic subjects, myocardial infarction survivors, and healthy adults. *Journal of Lipid Research*.

[39] Reymer PWA, Gagne E, Groenemeyer BE (1995). A lipoprotein lipase mutation (Asn291Ser) is associated with reduced HDL cholesterol levels in premature atherosclerosis. *Nature Genetics*.

[40] Chang YT, Chang MC, Su TC (2009). Lipoprotein lipase mutation S447X associated with pancreatic calcification and steatorrhea in hyperlipidemic pancreatitis. *Journal of Clinical Gastroenterology*.

[41] Nordestgaard BG, Abildgaard S, Wittrup HH, Steffensen R, Jensen G, Tybjærg-Hansen A (1997). Heterozygous lipoprotein lipase deficiency: frequency in the general population, effect on plasma lipid levels, and risk of ischemic heart disease. *Circulation*.

[42] Wilson DE, Emi M, Iverius PH (1990). Phenotypic expression of heterozygous lipoprotein lipase deficiency in the extended pedigree of a proband homozygous for a missense mutation. *Journal of Clinical Investigation*.

[43] Miesenbock G, Holzl B, Foger B (1993). Heterozygous lipoprotein lipase deficiency due to a missense mutation as the cause of impaired triglyceride tolerance with multiple lipoprotein abnormalities. *Journal of Clinical Investigation*.

[44] Semenkovich CF, Heinecke JW (1997). The mystery of diabetes and atherosclerosis: time for a new plot. *Diabetes*.

[45] Lu W, Takahashi H, Furusato B (2006). Allelotyping analysis at chromosome arm 8p of high-grade prostatic intraepithelial neoplasia and incidental, latent, and clinical prostate cancers. *Genes Chromosomes and Cancer*.

[46] Gallucci M, Merola R, Farsetti A (2006). Cytogenetic profiles as additional markers to pathological features in clinically localized prostate carcinoma. *Cancer Letters*.

[47] Bova GS, Carter BS, Bussemakers MJG (1993). Homozygous deletion and frequent allelic loss of chromosome 8p22 loci in human prostate cancer. *Cancer Research*.

[48] Xu J, Zheng SL, Hawkins GA (2001). Linkage and association studies of prostate cancer susceptibility: evidence for linkage at 8p22-23. *American Journal of Human Genetics*.

[49] Knowles MA, Aveyard JS, Taylor CF, Harnden P, Bass S (2005). Mutation analysis of the 8p candidate tumour suppressor genes DBC2 (RHOBTB2) and LZTS1 in bladder cancer. *Cancer Letters*.

[50] Seng TJ, Low JSW, Li H (2007). The major 8p22 tumor suppressor DLC1 is frequently silenced by methylation in both endemic and sporadic nasopharyngeal, esophageal, and cervical carcinomas, and inhibits tumor cell colony formation. *Oncogene*.

[51] Di Benedetto M, Pineau P, Nouet S (2006). Mutation analysis of the 8p22 candidate tumor suppressor gene ATIP/MTUS1 in hepatocellular carcinoma. *Molecular and Cellular Endocrinology*.

[52] Di Benedetto M, Bièche I, Deshayes F (2006). Structural organization and expression of human MTUS1, a candidate 8p22 tumor suppressor gene encoding a family of angiotensin II AT2 receptor-interacting proteins, ATIP. *Gene*.

[53] Narita S, Tsuchiya N, Wang L (2004). Association of lipoprotein lipase gene polymorphism with risk of prostate cancer in a Japanese population. *International Journal of Cancer*.

[54] Kim JW, Cheng Y, Liu W (2009). Genetic and epigenetic inactivation of LPL gene in human prostate cancer. *International Journal of Cancer*.

[55] Raju J, Bird RP (2003). Energy restriction reduces the number of advanced aberrant crypt foci and attenuates the expression of colonic transforming growth factor *β* and cyclooxygenase isoforms in Zucker obese (fa/fa) rats. *Cancer Research*.

[56] Ochiai M, Ogawa K, Wakabayashi K (1991). Induction of intestinal adenocarcinomas by 2-amino-1-methyl-6-phenylimidazo-[4,5-b]pyridine in Nagase analbuminemic rats. *Japanese Journal of Cancer Research*.

[57] Koohestani N, Tran TT, Lee W, Wolever TMS, Bruce WR (1997). Insulin resistance and promotion of aberrant crypt foci in the colons of rats on a high-fat diet. *Nutrition and Cancer*.

[58] Quesada CF, Kimata H, Mori M, Nishimura M, Tsuneyoshi T, Baba S (1998). Piroxicam and acarbose as chemopreventive agents for spontaneous intestinal adenomas in APC gene 1309 knockout mice. *Japanese Journal of Cancer Research*.

[59] Niho N, Takahashi M, Kitamura T (2003). Concomitant suppression of hyperlipidemia and intestinal polyp formation in Apc-deficient mice by peroxisome proliferator-activated receptor ligands. *Cancer Research*.

[60] Niho N, Takahashi M, Shoji Y (2003). Dose-dependent suppression of hyperlipidemia and intestinal polyp formation in Min mice by pioglitazone, a PPAR*γ* ligand. *Cancer Science*.

[61] Teraoka N, Mutoh M, Takasu S (2011). High susceptibility to azoxymethane-induced colorectal carcinogenesis in obese KK-A(y) mice. *International Journal of Cancer*.

[62] Yasui Y, Suzuki R, Miyamoto S (2007). A lipophilic statin, pitavastatin, suppresses inflammation-associated mouse colon carcinogenesis. *International Journal of Cancer*.

[63] Takeuchi Y, Takahashi M, Sakano K (2007). Suppression of N-nitrosobis(2-oxopropyl)amine-induced pancreatic carcinogenesis in hamsters by pioglitazone, a ligand of peroxisome proliferator-activated receptor *γ*. *Carcinogenesis*.

[64] Tanaka T, Kohno H, Suzuki R, Yamada Y, Sugie S, Mori H (2003). A novel inflammation-related mouse colon carcinogenesis model induced by azoxymethane and dextran sodium sulfate. *Cancer Science*.

[65] Niho N, Mutoh M, Takahashi M, Tsutsumi K, Sugimura T, Wakabayashi K (2005). Concurrent suppression of hyperlipidemia and intestinal polyp formation by NO-1886, increasing lipoprotein lipase activity in Min mice. *Proceedings of the National Academy of Sciences of the United States of America*.

[66] Niho N, Mutoh M, Komiya M, Ohta T, Sugimura T, Wakabayashi K (2007). Improvement of hyperlipidemia by indomethacin in Min mice. *International Journal of Cancer*.

[67] Tsutsumi K, Inoue Y, Shima A, Iwasaki K, Kawamura M, Murase T (1993). The novel compound NO-1886 increases lipoprotein lipase activity with resulting elevation of high density lipoprotein cholesterol, and long-term administration inhibits atherogenesis in the coronary arteries of rats with experimental atherosclerosis. *Journal of Clinical Investigation*.

[68] Doi M, Kondo Y, Tsutsumi K (2003). Lipoprotein lipase activator NO-1886 (ibrolipim) accelerates the mRNA expression in fatty acid oxidation-related enzymes in rat liver. *Metabolism*.

[69] Akasu T, Yokoyama T, Sugihara K, Fujita S, Moriya Y, Kakizoe T (2002). Peroral sustained-release indomethacin treatment for rectal adenomas in familial adenomatous polyposis: a pilot study. *Hepato-Gastroenterology*.

[70] Chiu CH, McEntee MF, Whelan J (2000). Discordant effect of aspirin and indomethacin on intestinal tumor burden in Apc(Min/+) mice. *Prostaglandins Leukotrienes and Essential Fatty Acids*.

[71] Kusunoki N, Yamazaki R, Kawai S (2002). Induction of apoptosis in rheumatoid synovial fibroblasts by celecoxib, but not by other selective cyclooxygenase 2 inhibitors. *Arthritis and Rheumatism*.

[72] Kota RS, Ramana CV, Tenorio FA, Enelow RI, Rutledge JC (2005). Differential effects of lipoprotein lipase on tumor necrosis factor-*α* and interferon-*γ*-mediated gene expression in human endothelial cells. *Journal of Biological Chemistry*.

[73] Yu J, Chu ESH, Hui AY (2007). Lipoprotein lipase activator ameliorates the severity of dietary steatohepatitis. *Biochemical and Biophysical Research Communications*.

[74] Lichtenstein L, Mattijssen F, De Wit NJ (2010). Angptl4 protects against severe proinflammatory effects of saturated fat by inhibiting fatty acid uptake into mesenteric lymph node macrophages. *Cell Metabolism*.

[75] Schwarz S, Hufnagel B, Dworak M, Klumpp S, Krieglstein J (2006). Protein phosphatase type 2C*α* and 2C*β* are involved in fatty acid-induced apoptosis of neuronal and endothelial cells. *Apoptosis*.

[76] Goldberg IJ, Merkel M (2001). Lipoprotein lipase: physiology, biochemistry, and molecular biology. *Frontiers in Bioscience*.

[77] Gruen R, Hietanen E, Greenwood MRC (1978). Increased adipose tissue lipoprotein lipase activity during the development of the genetically obese rat (fa/fa). *Metabolism*.

[78] Schwartz RS, Brunzell JD (1978). Increased adipose tissue lipoprotein lipase activity in moderately obese men after weight reduction. *The Lancet*.

[79] Ong JM, Kern PA (1989). Effect of feeding and obesity on lipoprotein lipase activity, immunoreactive protein, and messenger RNA levels in human adipose tissue. *Journal of Clinical Investigation*.

[80] Seip RL, Angelopoulos TJ, Semenkovich CF (1995). Exercise induces human lipoprotein lipase gene expression in skeletal muscle but not adipose tissue. *American Journal of Physiology*.

[81] Kusunoki M, Hara T, Tsutsumi K (2000). The lipoprotein lipase activator, NO-1886, suppresses fat accumulation and insulin resistance in rats fed a high-fat diet. *Diabetologia*.

